# *Kalanchoe ceratophylla* (Crassulaceae): The True Identity of Sidingin, a Medicinal Plant From Sumatra, Based on Morphological and Molecular Evidence

**DOI:** 10.1155/tswj/1922406

**Published:** 2026-06-29

**Authors:** Lamhot Parulian Manalu, Abdullah Bin Arif, Arifin Surya Dwipa Irsyam, Himawan Adinegoro, Maisaroh Maisaroh, Muhammad Rifqi Hariri, Iin Pertiwi Amin Husaini, Dian Rosleine, Astuti Astuti, Olivia Bunga Pongtuluran, Rohmah Luthfiyanti, Nenie Yustiningsih, Kokom Komariyah, Subandrio Subandrio, Wahyu Purwanto, Okta Nama Putra

**Affiliations:** ^1^ Research Center for Equipment Manufacturing Technology, National Research and Innovation Agency (BRIN), KST Habibie, Tangerang, Selatan, Indonesia, brin.go.id; ^2^ Research Center for Horticulture, National Research and Innovation Agency (BRIN), KST Habibie, Bogor, Indonesia, brin.go.id; ^3^ Herbarium Bandungense (FIPIA), School of Life Sciences and Technology, Institut Teknologi Bandung (ITB), Sumedang, West Java, Indonesia, itb.ac.id; ^4^ Research Center for Process Technology, National Research and Innovation Agency (BRIN), KST Habibie, Banten, Indonesia, brin.go.id; ^5^ Research Center for Biosystematics and Evolution, National Research and Innovation Agency (BRIN), Bogor, West Java, Indonesia, brin.go.id; ^6^ Research Center for Applied Botany, National Research and Innovation Agency (BRIN), Bogor, West Java, Indonesia, brin.go.id; ^7^ Bureau for Organization and Human Resources, National Research and Innovation Agency, Jakarta, Indonesia, brin.go.id; ^8^ Research Center for Pharmaceutical Ingredients and Traditional Medicine, National Research and Innovation Agency (BRIN), Bogor, West Java, Indonesia, brin.go.id; ^9^ Research Center for Behavioral and Circular Economics, National Research and Innovation Agency (BRIN), Jakarta, Indonesia, brin.go.id; ^10^ Research Center for Social Welfare, Village and Connectivity, National Research and Innovation Agency (BRIN), Jakarta, Indonesia, brin.go.id

**Keywords:** *Kalanchoe ceratophylla*, medicinal plant, molecular analysis, morphological analysis, sidingin

## Abstract

*Kalanchoe* species is a plant that contains secondary metabolites that are beneficial for human health. However, the systematics of species in the genus *Kalanchoe* are often debated because these plants have many variations that easily cross between species and can reproduce asexually with adventitious buds. Previous studies have revealed that *Kalanchoe ceratophylla* Haw. is often misidentified as *Kalanchoe laciniata*. Therefore, the reidentification of *K. ceratophylla* (Crassulaceae) as the correct identity of sidingin, a medicinal plant widely used in Sumatra, challenges the prior identification of the species as *K. laciniata*. This study is aimed at determining the true identity of sidingin, a medicinal plant locally used in Sumatra, Indonesia, and traditionally recognized as *K. laciniata*. Specimens of sidingin were collected from Sumatra and analyzed using morphological and molecular approaches to clarify its taxonomic classification. The morphological analysis revealed that sidingin exhibits pinnately lobed leaves and other distinct characteristics, which prompted comparison with documented descriptions of *Kalanchoe* species. Molecular analysis, accomplished through ITS sequencing, indicated a 94.60% identity match with *Kalanchoe miteja*, supporting the morphological findings. The resulting phylogenetic tree positioned sidingin within the same clade as commercially available samples of *K. ceratophylla*, suggesting a potential reclassification of sidingin as *K. ceratophylla*. This study provides new insights into the distribution of *Kalanchoe* species in Sumatra, documenting it as a new record only recently discovered in this region. Furthermore, through integrating morphological and molecular evidence, this study confirms that sidingin is *K. ceratophylla*.

## 1. Introduction

*Kalanchoe* Adans. (Crassulaceae) comprises a total of 174 species, primarily found in the Tropical and Subtropical Old World [1]. The genus encompasses numerous herbaceous species characterized by succulent leaves and clustered flowers. *Kalanchoe* has gained significant popularity in horticulture due to its attractive flowers, long‐lasting blooming period, ability to withstand drought, ease of cultivation, and vigorous growth [2]. *Kalanchoe* is capable of both sexual and asexual reproduction, with plantlets growing around the margin of its leaves. Their capacity for vegetative reproduction enables them to escape cultivation and establish themselves in the wild. Several ornamental *Kalanchoe* have been reported to naturalize in Africa and the Gulf of Guinea Islands [3, 4], Australia [5], Asia [6–8], Southern Europe [9], Northern America [10], and Southern America [11, 12].

Certain species of *Kalanchoe* have also been employed in Asian traditional medicine, namely, *Kalanchoe crenata* (Andrews) Haw., *Kalanchoe integra* (Medik.) Kuntze, *Kalanchoe laciniata* (L.) DC., and *Kalanchoe pinnata* (Lam.) Pers [13, 14]. In addition, *Kalanchoe* is also used as traditional medicine in the Neotropics. *K. crenata* (Andrews) Haw. and *K. pinnata* have been traditionally employed by the Brazilian population to treat abscesses, burns, inflammation, rheumatism, and wounds [15]. In Indonesia, the fresh leaves of *K. pinnata* and *Kalanchoe prolifera* are commonly applied externally as a poultice for wounds, burns, scalds, and fever [16, 17]. The genus *Kalanchoe* contains secondary metabolites that have been widely used as herbal medicines [18] to treat fever, boils, expectorants, tonsillitis, and burns [19]. This plant extract can inhibit the growth of *Salmonella typhi* which causes high fever and typhoid [20].

The systematics of species in the genus *Kalanchoe* are often debated because these plants have many variations that easily cross between species and can reproduce asexually with adventitious buds [21]. During our field study in Sumatra, we collected a medicinal plant species that belongs to the genus *Kalanchoe* from Aceh, Padang, and Riau. Previous researchers have identified the plant as *K. laciniata*, and it has been commonly known as sidingin in Sumatra [22, 23]. Nevertheless, this plant differs from *K. laciniata* by having leaves that are deeply pinnatipartite. According to Fu et al. [24] and Fernandes et al. [19], the true *K. laciniata* has leaf margins with shallow, irregular lobes. Previous studies revealed that *Kalanchoe ceratophylla* Haw. is frequently misidentified as *K. laciniata* [6, 19, 24]. Fu et al. [24] noted that *K. ceratophylla* has pinnately lobed leaves at the middle of the stem, which is similar to our Sumatran samples. The species identified as *K. laciniata* in Indonesia could potentially be classified as *K. ceratophylla*.

In addition to morphological identification, molecular identification is needed to improve the accuracy and validity of the identification results of an organism. Scientists have developed a DNA‐based organism identification technique called DNA barcoding. The DNA sequence used for molecular identification is called a DNA barcode, which is a short DNA sequence (around 700 bp) whose position in the organism′s genome is known and is used to identify an organism [25]. The source of the sequences used in DNA barcoding can be obtained from nuclear DNA (nDNA), chloroplast DNA (cpDNA), and mitochondrial DNA (mtDNA) [26]. The DNA barcoding technique was developed with the aim of accelerating and simplifying the process of molecular identification of an organism. This technique has several advantages, as it can be carried out on an organism in all forms of life stages, on organisms that are not intact, and on organisms whose DNA has been degraded, and can be applied to various species whose morphological forms are difficult to distinguish [25]. In practice, the convenience offered by the DNA barcoding technique must be accompanied by a complete DNA barcode sequence database and other related data, such as morphology and other phenotypes. This means that both identification techniques, conventional and molecular, are complementary [27]. Thus, the purpose of this study is to determine the identity of sidingin through morphological and molecular analysis.

## 2. Materials and Method

### 2.1. Plant Material Collection and Morphological Observation

Specimens of sidingin from the island of Sumatra, Indonesia, and those available on e‐commerce were collected according to van Balgooy′s [28] guidelines and preserved as dried and wet specimens following Bridson and Forman [29]. In addition, we collected living specimens and planted them in Cimanggis, Depok, West Java, Indonesia. Morphological observations were conducted at the Herbarium Bandungense (FIPIA), which is located at the School of Life Sciences and Technology, Institut Teknologi Bandung (ITB), as well as at the Laboratory of Gedung Utara, Bogor Botanic Gardens. Further herbarium study was carried out in the Herbarium Bogoriense (BO), FIPIA, and the Singapore Botanic Gardens Herbarium (SING). Specimens of sidingin were identified using taxonomic references on the genus *Kalanchoe*, including Wickens [26], Fu et al. [24], Wang et al. [6], and Smith et al. [30].

### 2.2. Molecular Analysis

Leaves from two samples of sidingin, one sample from Riau (*Maisaroh s.n.*) and the other from e‐commerce, were reduced to powder with a mortar and pestle. Total DNA was extracted using the standard Tiangen Plant DNA Extraction Kit (Tiangen Biotech, Beijing). Sun et al. [31] described the use of 17SE and 26SE ITS primers for PCR amplification. The PCR reaction used 25 *μ*L of MyTaq Master Mix (Bioline), 10 ng/*μ*L DNA, 5 *μ*M forward and reverse primers, and 11 *μ*L ddH_2_O. The PCR was performed in the following steps: initial denaturation at 94°C for 5 min, followed by 35 cycles of denaturation at 94°C for 30 s, primer annealing at 58°C for 30 s, and extension at 72°C for 30 s. The last extension stage was carried out for 5 min at 72°C.

PCR products were electrophoresed on a 1% agarose gel in a 1× TAE buffer containing 1 *μ*L FloroSafe. DNA purification and sequencing were performed at 1st BASE (Malaysia) using a Sanger sequencing machine. Delivery of PCR products and sequencing services was assisted through PT Genetika Science Indonesia.

To minimize nucleotide reading bias, the forward and reverse sequences were edited, and the chromatograms were confirmed. Both sequences were assembled into contigs and compared using the National Center for Biotechnology Information (NCBI, http://www.ncbi.nml.nih.gov) database, through the basic local alignment search tool (BLAST) option. To validate the taxonomic classification of the analyzed samples, NCBI sequences of other *Kalanchoe* species taken from the NCBI database were included together in a phylogenetic analysis, using *Hylotelephium erythrostictum* (Miq.) H.Ohba (Accession Number JQ954558.1) as the outgroup. All sequences were aligned using the Clustal W algorithm and saved in FASTA format for subsequent phylogenetic analysis. The phylogenetic tree was constructed using the maximum likelihood (ML) method, with the Tamura 3‐parameter substitution model applied to account for evolutionary rate differences among sites. A discrete gamma distribution was used to model rate variation (five categories [+G, parameter = 0.4891]), and the robustness of the tree topology was assessed using 1000 bootstrap replicates. All analyses were conducted using MEGA 11 software [32], ensuring a rigorous and reproducible approach to phylogenetic inference.

## 3. Result and Discussion

### 3.1. Morphology Identification of Sidingin

**Figure 1:**
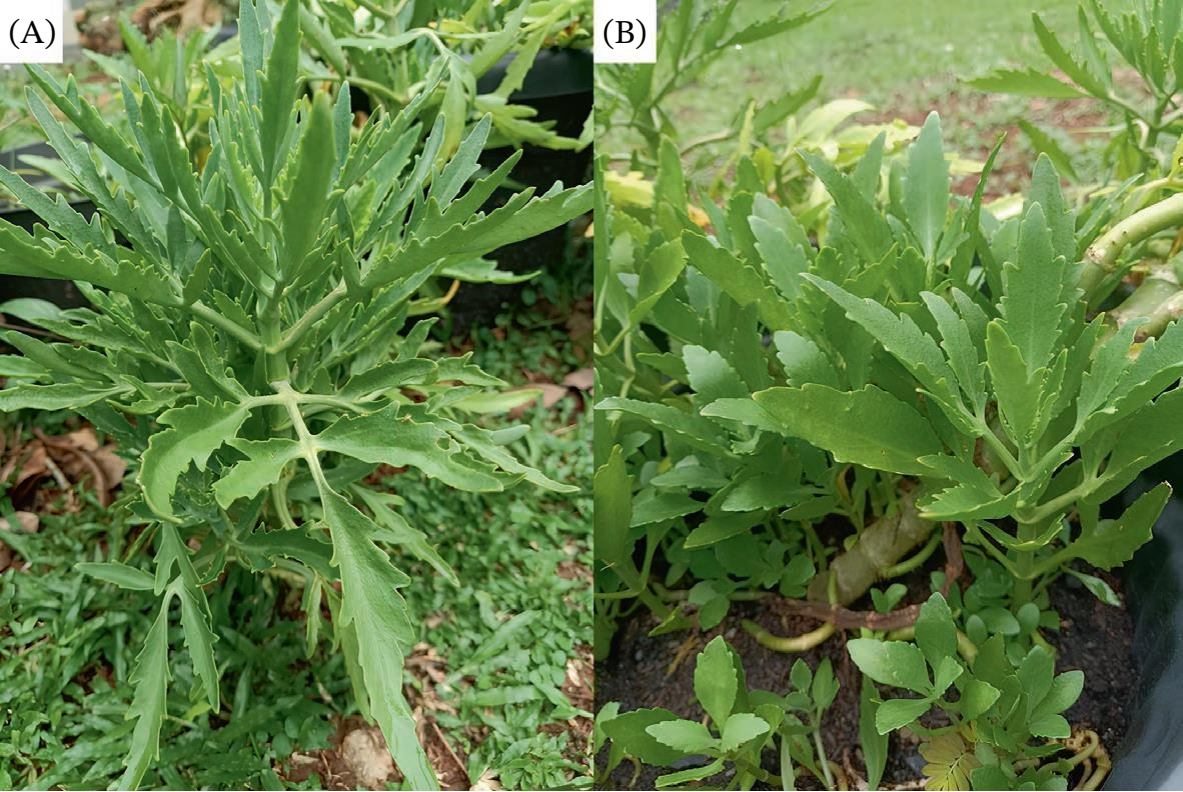
*Kalanchoe ceratophylla*. (A) Adult plant. (B) Juvenile plants. The figure was taken by the authors.

The specimens collected from Sumatra were identified as *K. ceratophylla* based on diagnostic morphological characters. The identification was supported by the presence of pinnately lobed leaves with ovate to lanceolate lobes, irregularly serrate margins, and acute apices in all examined samples (Figure 1). These characters correspond to *K. ceratophylla* as described by Wickens [26] and Fu et al. [24] and clearly differ from *K. laciniata*, which has shallowly and irregularly lobed leaf margins [19, 24]. Therefore, the evidence indicates that earlier reports of sidingin as *K. laciniata* represent misidentifications rather than evidence that *K. ceratophylla* and *K. laciniata* are the same species. A diagnostic comparison is provided in Table 1.

**Table 1:** Diagnostic morphological comparison between *Kalanchoe ceratophylla* and *Kalanchoe laciniata* used for identification of sidingin.

Character	*K. ceratophylla*	*K. laciniata*	Sumatran sidingin
Leaf division	Deeply pinnately lobed to pinnatipartite, commonly 3–7‐lobed	Shallowly and irregularly lobed	Deeply pinnately lobed, 3–7‐lobed
Lobe shape	Ovate to lanceolate lobes	Lobes less deeply divided and irregular	Ovate to lanceolate lobes
Leaf margin and apex	Margins irregularly to coarsely serrate; apex acute	Margins shallowly irregularly lobed; apex not matching the Sumatran material	Margins irregularly serrate; apex acute
Taxonomic interpretation	Matches the diagnostic morphology reported by Wickens [26] and Fu et al. [24]	Does not match the Sumatran material as described by Fu et al. [24] and Fernandes et al. [19]	Identified as *K. ceratophylla*; previous use of *K. laciniata* is treated as a misapplied name

Herb or subshrub, erect to ascending, up to 34 cm tall. Stem terete, 1 cm diam., glabrous, green; internodes 1.2–5 cm long. Leaves opposite, pinnately lobed, 3–7‐lobed; petiole curved, 2.8–4 cm long, canaliculate, wider at base; lobes ovate to lanceolate (5–10 × 1.5–4.5 cm), base cuneate to attenuate, margin irregularly serrate, apex acute, adaxial surface green, abaxial surface grayish green. Flowers and fruits were not observed.

*K. ceratophylla* Haw., *Revis. Pl. Succ.* 23 (1821); Wickens, *Kew Bull.* 36(4): 673 (1982). *Vereia ceratophylla* (Haw.) D. Dietr., *Syn. Pl.* 2: 1328 (1840).

Type: CHINA. Cultivated at Kew from material originating from China; holotype: illustration by T. Duncanson, c. 1822–1826 (K), cited by Wickens [26].

Distribution: The species has a natural range that extends from the Indian subcontinent to Southern China and Indochina, including Taiwan [1]. Wickens [26] states that the species has been intentionally grown in areas outside its original geographic range, notably Hong Kong, the Philippines, Java, and the Mascarenes. In our study, *K. ceratophylla* was collected from several locations in Sumatra, including Aceh and Riau provinces. Its presence in Sumatra has not previously been documented.

Specimens examined: INDONESIA. Sumatra: Aceh, Banda Aceh, Syiah Kuala Subdistrict, Pineung, Jl. Tgk. Chik Dipineung Raya No. 3, 1.VIII.2024, *M.M. Asyawari s.n.* (FIPIA); Riau, Pulau Rangsang, October 1930, *Z. Teruya 1573* (SING‐0200043). Java: Cultivated in Depok from material collected in Riau (ex Sumatra: Pekanbaru City, Bukit Raya Subdistrict, Simpang Tiga, original collection December 2022, *Ria Aulia & Eliwarti s.n.*), voucher 24.VII.2024, *Maisaroh s.n.* (FIPIA).

Vernacular names: Sisijuk (Aceh), sidingin (Padang, Riau).

Uses: Medicinal plant.

*K. ceratophylla* has recently been identified as a new record in the flora of Sumatra. Previously, this species was only documented in Java [26], and its existence in Sumatra had not been noted by other botanists. Taxonomic information regarding *Kalanchoe* in Sumatra is limited, as no species of this genus are naturally distributed on the island [1]. The only recognized species commonly cultivated in Sumatra is *K. pinnata*, which was introduced from Madagascar [1]. Consequently, this study provides the most up‐to‐date taxonomic information on *Kalanchoe* in Sumatra, increasing the number of cultivated *Kalanchoe* species on the island to two species.

*K. ceratophylla* may have been introduced to Sumatra through the trade of ornamental plants. Nevertheless, the precise origins of its introduction in Sumatra remain uncertain. Based on the specimen examination conducted by the SITH ITB team, the earliest record of *K. ceratophylla* in Sumatra may be traced back to the 20th century, as observed from a specimen in the SING Herbarium. The specimen SING 0200043 was collected from Rangsang Island, Riau, in October 1930 by Z. Teruya. The plant was cultivated as an ornamental by the local people on Rangsang Island. *K. ceratophylla* is currently extensively cultivated for ornamental uses in Aceh, West Sumatra, and Riau. This plant is known as sidingin or sisijuk due to its ability to produce a refreshing effect when the leaves are crushed and applied topically.

*K. ceratophylla* is used as a traditional medicine to reduce fever in children. The plant is believed to possess anti‐inflammatory, antibacterial, and antioxidant properties. According to our study, this plant has been used to treat minor wounds, burns, or insect bites by crushing its leaves and applying them to the affected area. The leaves are traditionally used by the people of Riau, Indonesia, and surrounding areas as a natural remedy and an antipyretic agent through a straightforward method. It is a traditional way to help children feel better when they have a fever without using chemical medicine. The process involves finding fresh leaves from the sidingin plant and then washing the fresh leaves thoroughly to ensure cleanliness. Next, soak the leaves in water and gently squeeze them to release “active ingredients” believed to have antipyretic (fever‐reducing) properties. These are the unique parts of the plant that help bring down the fever. Furthermore, the mixture of water and squeezed leaves is applied gently to the body′s essential spots, such as the forehead, under the arms (armpits), and the back places that help control body temperature. In the last stage, this process might be done a few times if the fever is high, but after doing it two to three times, the child starts feeling cooler, typically resulting in a decrease in body temperature after two to three applications.

### 3.2. Molecular Identification of Sidingin

**Figure 2:**
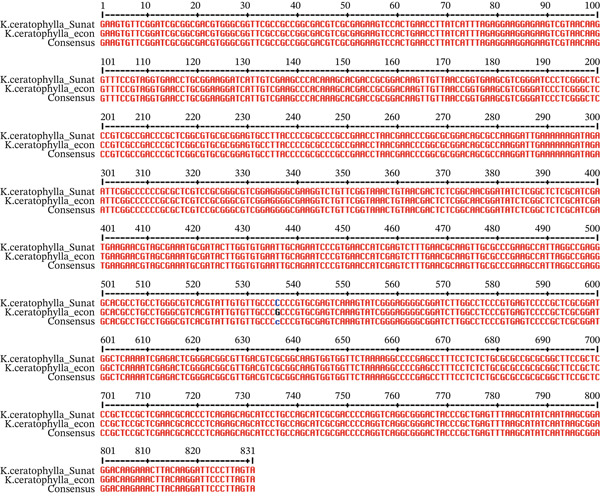
Nucleotide comparison between *Kalanchoe ceratophylla* from Sumatra and the sample sold in e‐commerce.

The specimens collected from Sumatra (*Maisaroh s.n.*) and obtained from e‐commerce are clearly identified as *K. ceratophylla* through morphological analysis. In the analysis of the 100 hits from the BLAST results, *K. ceratophylla* exhibited a 94.60% identity match with *Kalanchoe miteja*. Notably, 84% of the query comprised fragments of the 5.8S ribosomal RNA gene, the complete sequence of internal transcribed Spacer 2 (ITS2), and fragments of the large subunit ribosomal RNA gene, as there is currently no database sequence available for *K. ceratophylla* in NCBI. Furthermore, the sequence of *K. ceratophylla* from Sumatra differs by only one nucleotide (Site 536) from *K. ceratophylla* available in e‐commerce (Figure 2), indicating that sidingin is often incorrectly labeled as *K. laciniata* instead of *K. ceratophylla* [6, 24, 26], a common error made by horticulturists.

Given the long‐standing taxonomic ambiguity between *K. ceratophylla* and *K. laciniata*, it is necessary to include *K. laciniata* in the phylogenetic reconstruction to clarify their evolutionary relationships within the genus. Historically, specimens of *K. ceratophylla* have often been misidentified as *K. laciniata* due to similarities in leaf dissection and overall vegetative morphology [19, 24]. However, *K. ceratophylla* possesses distinctly pinnately lobed leaves with deeper and more regular lobation compared to the shallowly irregularly lobed leaves of *K. laciniata.* The inclusion of *K. laciniata* sequences in the molecular analysis would enable a direct comparison of both taxa, providing molecular evidence to support or refute their close relationship. Such comparative analysis would not only strengthen the reidentification of sidingin as *K. ceratophylla* but also help determine whether the morphological resemblance between the two taxa results from shared ancestry or convergent evolution. Therefore, incorporating *K. laciniata* in future phylogenetic analyses is essential to resolve historical misidentifications and to refine the infrageneric taxonomy of *Kalanchoe*.

**Figure 3:**
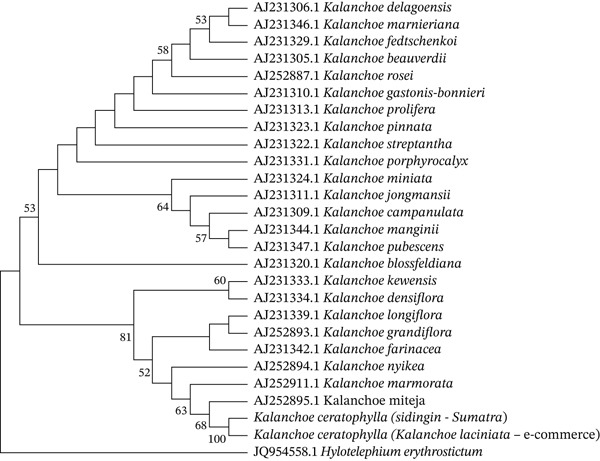
ITS‐based phylogenetic placement of the two analyzed *K. ceratophylla* samples. The topology supports the close relationship between the Sumatran and e‐commerce samples but does not constitute a full species boundary test with *K. laciniata*.

The phylogenetic tree with the highest log likelihood (−3904.61) provided preliminary support for the morphological identification. The Sumatran and e‐commerce samples of *K. ceratophylla* were grouped in the same clade with a bootstrap value of 100, indicating that both analyzed specimens are closely related and likely belong to the same taxon (Figure 3). However, because authenticated sequences of *K. laciniata* from different populations were not included, the tree should not be interpreted as a definitive test of whether *K. ceratophylla* and *K. laciniata* are conspecific or distinct. *Kalanchoe* is one of the most diverse and taxonomically complex genera, and its identification remains challenging for field workers [33]. The use of ITS can support taxonomic assessment, but reliable species delimitation requires integration with voucher‐based morphology and broader sampling [34].

Overall, the classification of sidingin as *K. ceratophylla* is based primarily on diagnostic morphological congruence, with ITS data providing preliminary support. The absence of authenticated, vouchered *K. laciniata* sequences prevents direct molecular comparison in the present dataset. Therefore, the molecular evidence should be viewed as supplementary to morphology rather than as stand‐alone proof of species delimitation within this complex.

Subsequent research using verified specimens of *K. laciniata* and *K. ceratophylla* from multiple populations, together with additional molecular markers such as chloroplast regions, will be essential to clarify phylogenetic relationships and unequivocally delineate species boundaries within this complex.

### 3.3. Taxonomic Treatment

#### 3.3.1. Key to the Genus *Kalanchoe* of Sumatra

A. Leaves entire to pinnately lobed, lobes oblong to elliptic, margin crenate, apex obtuse: *K. pinnata*

B. Leaves pinnately lobed, lobes ovate to lanceolate, margin coarsely serrate, apex acute: *K. ceratophylla*

Population‐level sampling represents an important approach in plant taxonomy for assessing intraspecific variation and species limits. The present study included specimens collected from multiple localities in Sumatra and examination of historical herbarium material; however, it was not designed as a comprehensive population genetic study. The diagnostic morphological characters observed across all examined specimens were consistent and stable, supporting their placement within *K. ceratophylla*. Nevertheless, broader geographic sampling combined with morphometric and multilocus molecular analyses would provide a more detailed understanding of variation patterns and evolutionary relationships within Southeast Asian populations of *Kalanchoe*. Such integrative studies are strongly encouraged for future study.

## 4. Conclusion

This study identifies sidingin as *K. ceratophylla* primarily on the basis of diagnostic morphology, supported by preliminary ITS evidence showing that the Sumatran and e‐commerce samples are nearly identical. The study does not establish *K. ceratophylla* and *K. laciniata* as conspecific; rather, it clarifies that previous records of sidingin under the name *K. laciniata* are likely misidentifications. Because authenticated *K. laciniata* sequences and population‐level sampling were not available, future studies should include multiple populations of both taxa and additional molecular markers. The present record of *K. ceratophylla* from Sumatra enriches the taxonomic understanding of cultivated *Kalanchoe* in Indonesia and highlights the importance of accurate identification for medicinal plant documentation.

## Author Contributions

**Lamhot Parulian Manalu** contributed to conceptualization, funding acquisition, methodology, investigation, visualization, supervision, writing—original draft, and writing—review and editing. **Abdullah Bin Arif** contributed to conceptualization, formal analysis, methodology, investigation, visualization, writing—original draft, and writing—review and editing. **Arifin Surya Dwipa Irsyam** contributed to conceptualization, methodology, investigation, visualization, software, writing—original draft, and writing—review and editing. **Himawan Adinegoro** contributed to conceptualization, methodology, investigation, visualization, writing—original draft, and writing—review and editing. **Maisaroh Maisaroh** contributed to investigation, data curation, resources, and writing—original draft. **Muhammad Rifqi Hariri** contributed to data curation, visualization, validation, formal analysis, software, and writing—original draft. **Iin Pertiwi Amin Husaini** contributed to data curation, formal analysis, visualization, and validation. **Dian Rosleine** contributed to data curation, visualization, validation, and software. **Astuti Astuti** contributed to project administration, investigation, data curation, resources, and writing—original draft. **Olivia Bunga Pongtuluran** contributed to the investigation and data curation. **Rohmah Luthfiyanti** contributed to the investigation and data curation. **Nenie Yustiningsih** contributed to data curation, formal analysis, visualization, validation, and writing—original draft. **Kokom Komariyah** contributed to resources and data curation. **Subandrio Subandrio** contributed to conceptualization, methodology, investigation, visualization, writing—original draft, and writing—review and editing. **Wahyu Purwanto** contributed to data curation, visualization, validation, and writing—original draft. **Okta Nama Putra** contributed to the investigation and data curation.

## Funding

This work was supported by the Indonesia Endowment Fund for Education Agency (LPDP), Ministry of Finance, and the National Research and Innovation Agency (BRIN), Indonesia (under the RIIM‐4 Grant Nos. B‐3844/II.7.5/FR.06.00/11/2023 and B‐4113/III.11/PR.02/11/2023).

## Conflicts of Interest

The authors declare no conflicts of interest.

## Data Availability

The data used to support the findings of this study are available from the corresponding authors upon request.
